# Oxidative Phosphorylation in Silent Pituitary Adenomas: A Multiomics Perspective

**DOI:** 10.1155/ije/8488950

**Published:** 2026-01-28

**Authors:** Yuan Chen, Qiang Zhao, Xing Wang, Xinling Wang, Yanying Guo

**Affiliations:** ^1^ Department of Endocrinology and Metabolic Diseases, People’s Hospital of Xinjiang Uygur Autonomous Region, Xinjiang Clinical Research Center for Diabetes, Urumqi, Xinjiang, China; ^2^ Heart and Pan-vascular Medical Diagnosis and Treatment Center, People’s Hospital of Xinjiang Uygur Autonomous Region, Urumqi, Xinjiang, China, xjrmyy.com; ^3^ Xinjiang Key Laboratory of Cardiovascular Homeostasis and Regeneration Research, People’s Hospital of Xinjiang Uygur Autonomous Region, Urumqi, Xinjiang, China, xjrmyy.com

## Abstract

Although the World Health Organization has clearly defined silent pituitary adenomas (SPAs) and functional adenomas (non‐SPAs), the detailed biological mechanisms remain unclear. This study conducted a comprehensive analysis of clinical, genomic, transcriptomic, and proteomic differences between SPA and non‐SPA. The results revealed significant differences in mutational profiles, with a notably higher mutation rate of the TCHH gene in non‐SPA samples. Transcriptomic and proteomic analyses identified distinct expression patterns, highlighting the enrichment of the oxidative phosphorylation pathway and other related Gene Ontology terms in SPA samples. To validate these findings, 11 additional pituitary adenoma samples were analyzed, confirming the critical role of oxidative phosphorylation in SPA. Activation of oxidative phosphorylation altered the hormone secretion, and the electron transfer chain inhibitor restored this in both human and rat pituitary adenoma cell lines. Furthermore, a protein–protein interaction network was constructed, revealing key regulatory differences between SPA and non‐SPA, and identifying MAPK1, MAPK3, IDH1, and PKM as key hubs in the network. MAPK1 and PKM knockdown significantly reduced the hormone secretion and apoptosis of both cell lines. These findings suggest that the oxidative phosphorylation pathway plays a pivotal role in the secretory functions of pituitary adenomas. This study offers new insights into the biological mechanisms underlying pituitary adenomas and provides valuable directions for future research, emphasizing the importance of oxidative phosphorylation in tumor behavior and potential therapeutic targets.

## 1. Introduction

Pituitary adenomas, also called pituitary neuroendocrine tumors, are highly heterogeneous due to their different cell type origins [1]. According to the World Health Organization’s (WHO) classification rules, the originating cell lineages are classified into lactotroph, thyrotroph, corticotroph, gonadotroph, null cell, and pituitary‐specific positive transcription factor (TF) 1 (PIT1)–positive adenoma [2]. Different types have distinct hormone secretion profiles and TF activations. For example, somatotroph adenomas secrete the alpha subunit of GH and activate PIT1, while lactotroph adenomas secrete prolactin (PRL) by activating PIT1 and ERα [3]. Based on their secretory functions, pituitary adenomas are classified into null cell, silent, clinically silent, whispering, and functioning adenomas [3, 4]. “Silent pituitary adenoma” (SPA) means that these tumors express more than one hormone and/or TF, but clinically, these hormones are not secreted [5, 6]. There is also another subtype, null cell adenoma, which does not express any hormones or TFs and can transform into other subtypes, including SPA. Thus, understanding the pituitary process is critical for comprehending the biological mechanisms of pituitary adenomas.

A study involving 1065 pituitary adenomas revealed that most were silent (84%), and most of the TSH‐staining samples were plurihormonal, especially those expressing GH [7]. Recently, single‐cell sequencing showed that the function of PIT1‐positive (functional) adenomas was associated with carcinogenesis, which is linked to INF‐γ that induces the remodeling of cancer‐associated fibroblasts (CAFs). This axis also involves N‐cadherin and STAT [8]. Gene expression of SPA and non‐SPA adenomas also differed. For example, decreased expression of P16, FGFR2, and GADD45γ was reported in pituitary adenomas, particularly in the SPA subtype [9].

In this study, we comprehensively analyzed the clinical, genomic, transcriptomic, and proteomic differences between SPA and non‐SPA samples. We also verified the results using 11 pituitary adenoma samples collected from our institution. We highlighted the role of the oxidative phosphorylation pathway in non‐SPA samples and provided insights into pituitary adenoma investigation.

## 2. Results

### 2.1. Clinical Association of Secretion and Clinical Indicators

The schema of this work is described in Figure 1(a). This study analyzed a publicly available dataset consisting of 200 pituitary adenoma samples with genomic mutation, gene expression, and proteomic data and validated it with 11 retrospectively collected samples. The association between clinical indicators (immune subtypes, gender, age, Ki67 ratio, and tumor volume) and secretion phenotype was examined. First, we divided the samples using age 60 and volume 5 cm^3^ as thresholds and analyzed the secretion and categories (Table S1). Secretion is significantly associated with immune subtype (*p* < 0.001). Immune‐exhausted pituitary adenomas tend to secrete different hormones, while CD8+ T cell infiltration and endothelial subtype tend to be SPA (Figure 1(b)). In addition, tumor volume was significantly associated with secretion subtypes, with pituitary adenomas that secrete hormones having a higher proportion of larger volumes (> 5 cm^3^). Although the categorized age (60 as a threshold) was not significantly associated with secretion phenotype, Student’s *t*‐test revealed that patients with SPA were significantly older than those with secretion (Figure 1(c)). On the other hand, the Ki67 ratio and gender (> 3%) were not significantly correlated with secretion phenotypes (Figures 1(d) and 1(e)). Collectively, these results indicate that secretion phenotype is associated with age, tumor size, and immune subtypes.

Figure 1Clinical association of secretion and clinical indicators in pituitary adenomas. (a) The schema of the study work analyzing pituitary adenoma samples. (b) Immune subtype association with secretion phenotype, showing a significant association (*p* < 0.001). (c) Age distribution between SPA and non‐SPA patients, with SPA patients being significantly older (*p* = 0.00023). (d) Gender distribution in SPA and non‐SPA groups, showing no significant difference (*p* = 1). (e) Ki67 ratio distribution in SPA and non‐SPA groups, showing no significant difference (*p* = 0.22).

**Figure:**
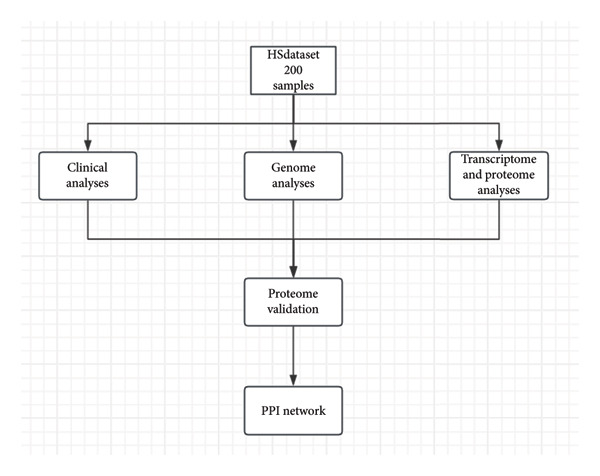
(a)

**Figure:**
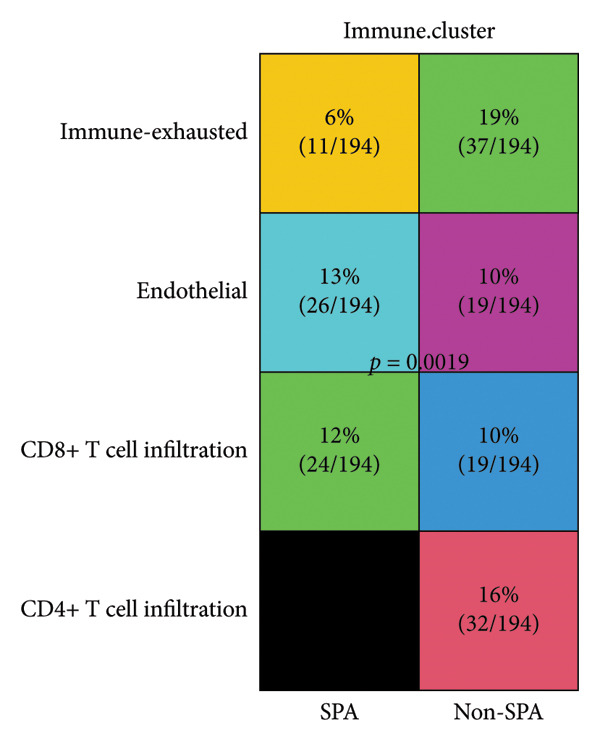
(b)

**Figure:**
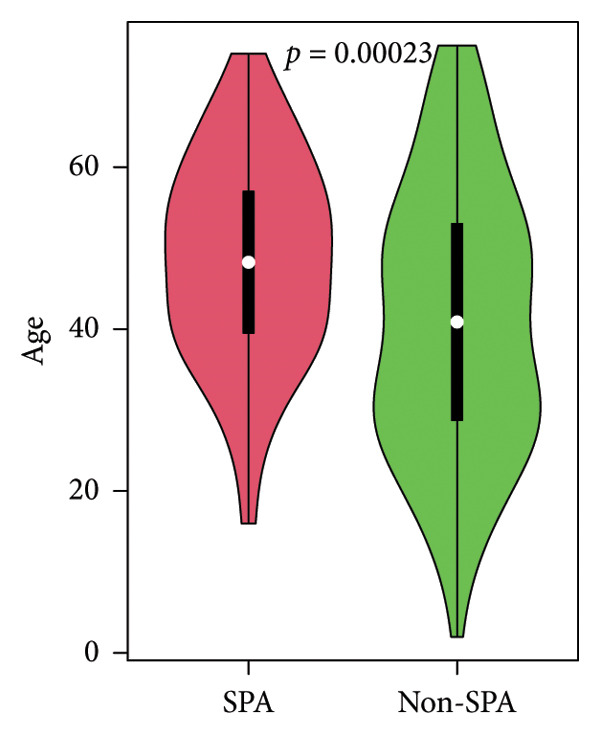
(c)

**Figure:**
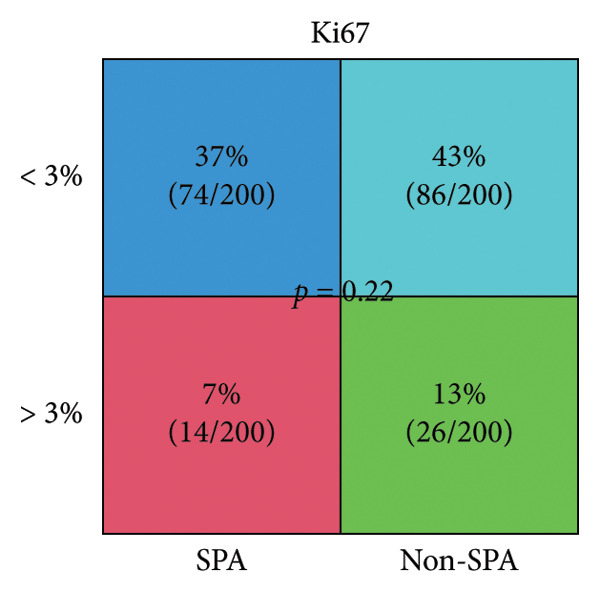
(d)

**Figure:**
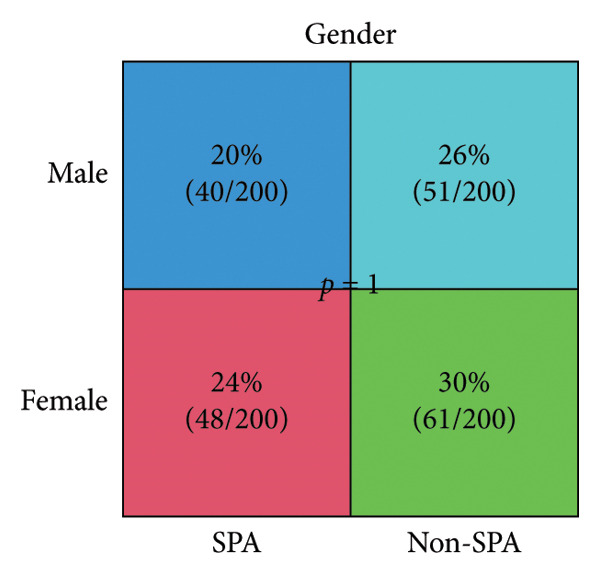
(e)

## 3. Genomic Signature of Secretion Phenotype

The genomic mutation differences between SPA and non‐SPA groups were identified. As shown in Figure 2(a), the mutational profiles of SPA and non‐SPA subgroups were similar, except for TCHH (Figure 2(b)). TCHH is mutated in 11 non‐SPA samples but only 2 SPA samples (*p* < 0.05), indicating differential mutation. The mutation of TCHH was sparse across the amino acid sequence, except for a p. 547–576 deletion and a p. 556–585 deletion. These sites were most distributed on the surface of the protein (Figure 2(c)) according to AlphaFold prediction. The observed mutation pattern, with dispersed sites frequently altering glutamic acid (E, 3/13, and 5/12 samples also contain E deletion) and arginine (R, 3/13, and 5/12 samples also contain E deletion), strongly suggests this gene is under positive selection pressure in cancer. These charged amino acids are often critical for forming salt bridges that stabilize protein structure or mediate key interactions, such as ligand binding. We also analyzed the correlation between immune infiltration and TCHH mutation. As a result, it was significantly associated with various cell types, including CD8+ T, natural killer (NK), γδT cells, and macrophages (Figure S1). In addition to the single gene mutation pattern, pathway‐level mutation differences between SPA and non‐SPA samples were analyzed. As shown in Figure 2(d), the ERBB, Hedgehog, homologous recombination, B cell receptor, and calcium signaling pathways were significantly differentially mutated. Collectively, these results indicate that the SPA phenotype is associated with TCHH and various pathway mutations.

Figure 2Genomic signature of secretion phenotype in pituitary adenomas. (a) Mutation landscape of SPA and non‐SPA samples. (b) Differential mutation rate of TCHH between SPA and non‐SPA samples (*p* < 0.05). (c) Distribution of TCHH mutation sites on AlphaFold‐predicted 3D structure. (d) Pathway‐level mutation differences between SPA and non‐SPA samples in ERBB, Hedgehog, homologous recombination, B cell receptor, and calcium signaling pathways.

**Figure:**
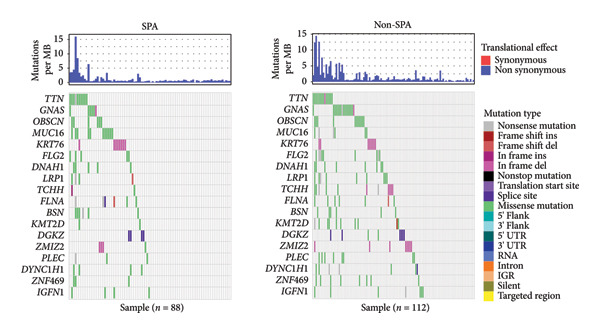
(a)

**Figure:**
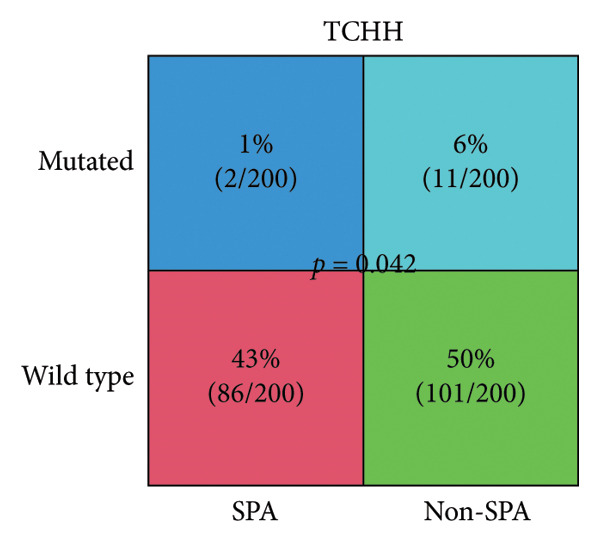
(b)

**Figure:**
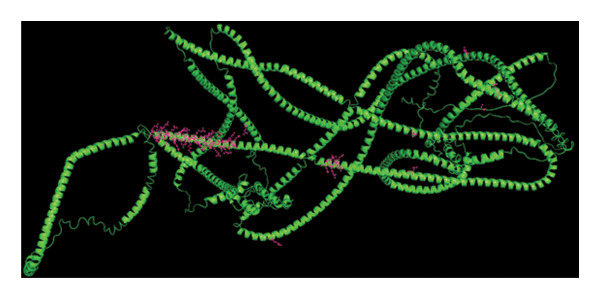
(c)

**Figure:**
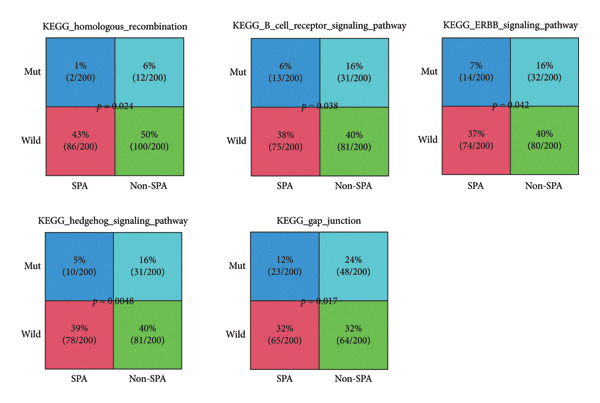
(d)

### 3.1. Transcriptomic and Proteomic Analyses Emphasized Oxidative Phosphorylation Pathway in Pituitary Adenoma Secretion

In addition to genomic signatures, transcriptomic and proteomic signatures of SPA were also identified. The differentially expressed genes and proteins were identified, as shown in Figures 3(a) and 3(b). These mRNAs and proteins clearly distinguished the SPA and non‐SPA samples (Figures 3(c) and 3(d)). Genes identified as both differentially expressed mRNA and proteins were intersected and used for Gene Ontology analyses. The enriched GO terms included protein‐processing pathways (e.g., Golgi vesicle transport) and GTPase regulator activity (Figures 3(e) and 3(f)). In addition, gene set enrichment analyses (GSEAs) were also implemented using both differentially expressed genes and proteins. As a result, 16 and 41 canonical KEGG signaling pathways were enriched, among which five pathways intersected, including cytokine–cytokine receptor interaction, neuroactive ligand receptor interaction, JAK‐STAT signaling, Parkinson’s disease, and oxidative phosphorylation signaling pathway (Figure 3(g)). Notably, the oxidative phosphorylation signaling pathway was significantly enriched in SPA samples instead of non‐SPA (Figures 3(h), 3(i)) in both transcriptomic and proteomic datasets. Taken together, these results indicate that the oxidative phosphorylation signaling pathway may play important roles in pituitary adenoma secretion.

Figure 3Transcriptomic and proteomic analyses in pituitary adenoma secretion. (a) Differentially expressed genes between SPA and non‐SPA samples. (b) Differentially expressed proteins between SPA and non‐SPA samples. (c) Clustering of samples based on mRNA expression profiles. (d) Clustering of samples based on protein expression profiles. (e) Enriched Gene Ontology (biological process) terms in differentially expressed genes and proteins. (f) Enriched Gene Ontology (molecular function) terms in differentially expressed genes and proteins. (g) Gene set enrichment analyses (GSEA) showing significantly enriched KEGG signaling pathways based on protein and mRNA. (h) Enrichment of oxidative phosphorylation signaling pathway in SPA samples based on transcriptome. (i) Enrichment of oxidative phosphorylation signaling pathway in SPA samples based on proteome.

**Figure:**
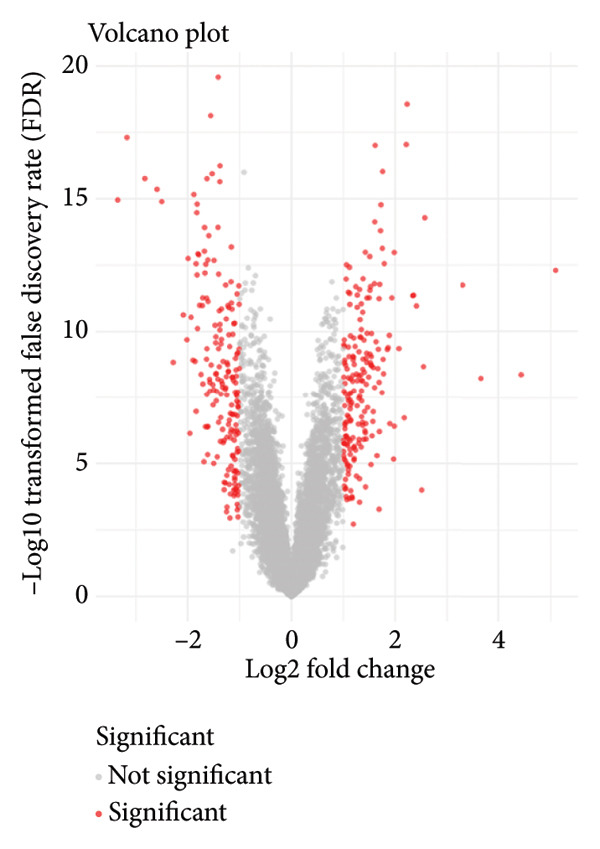
(a)

**Figure:**
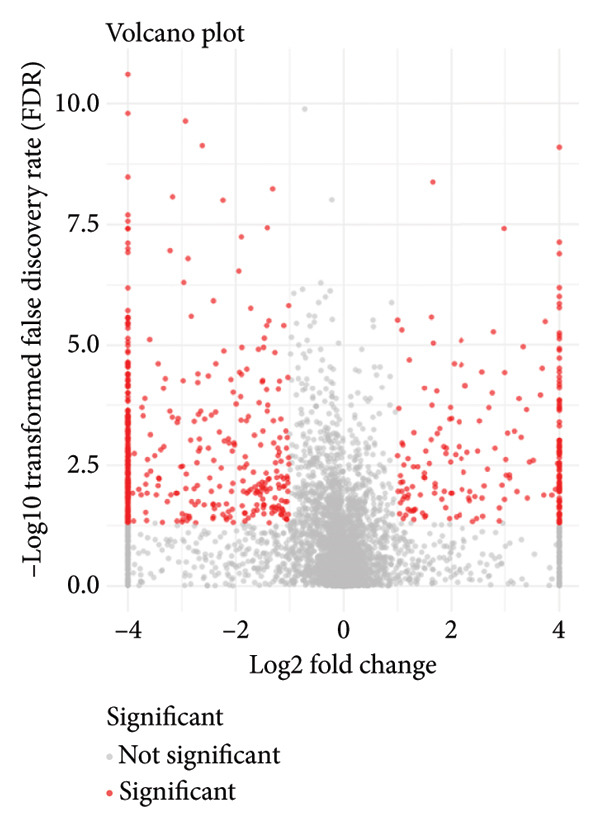
(b)

**Figure:**
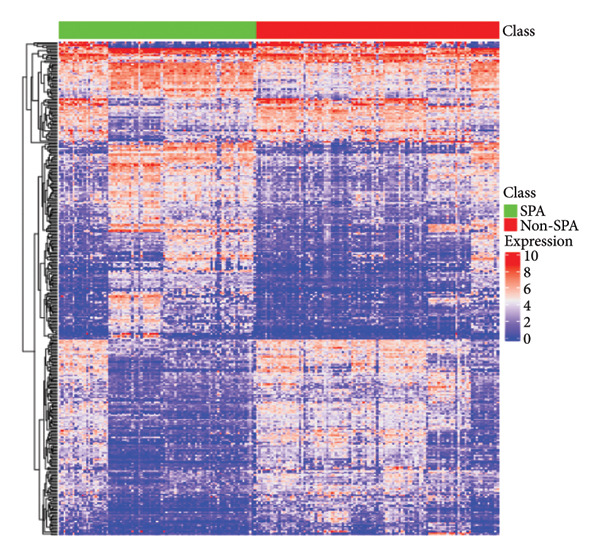
(c)

**Figure:**
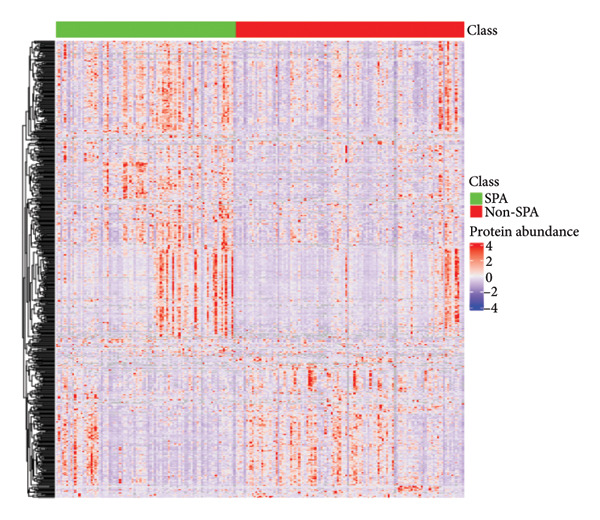
(d)

**Figure:**
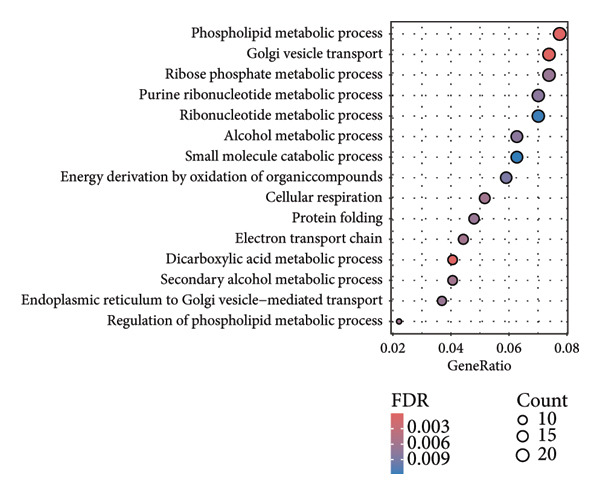
(e)

**Figure:**
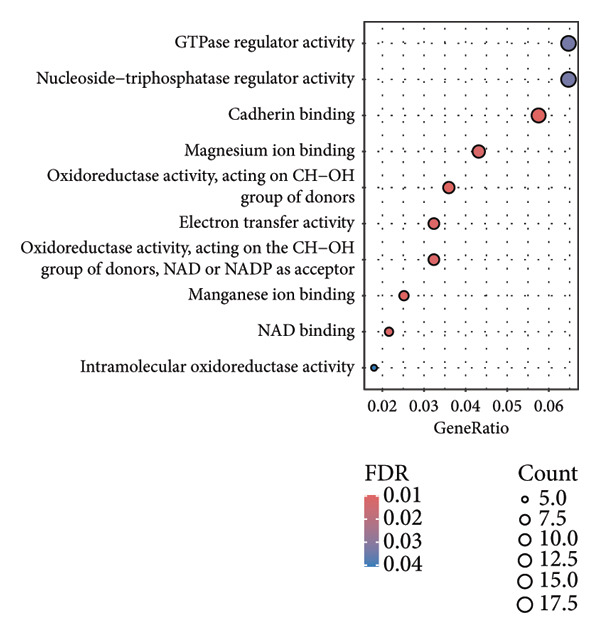
(f)

**Figure:**
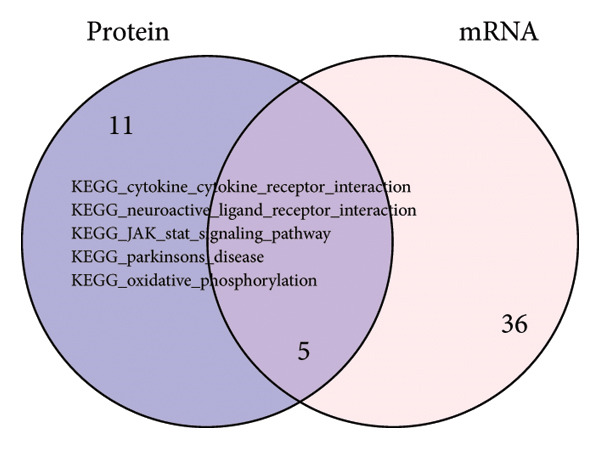
(g)

**Figure:**
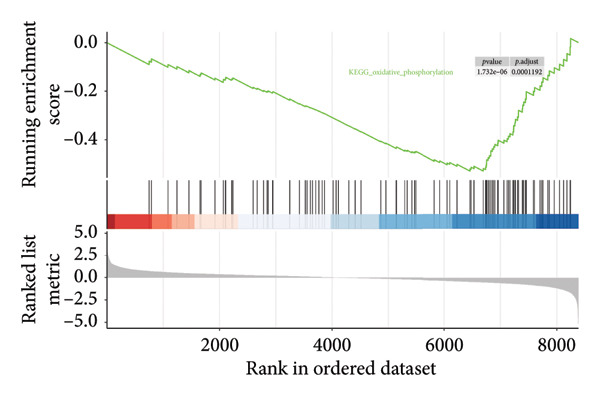
(h)

**Figure:**
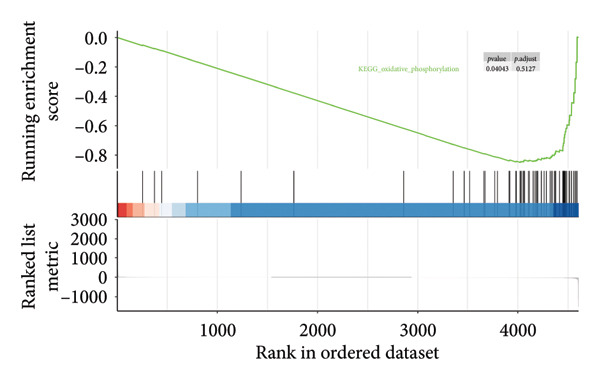
(i)

### 3.2. Oxidative Phosphorylation Pathway in the Validation Proteomic Dataset

Since proteomics has been widely applied in disease‐related studies [10], to verify the role of the oxidative phosphorylation pathway in pituitary adenoma secretion, we prospectively collected 11 pituitary adenoma samples (five non‐SPA and six SPA) and conducted nontargeted proteome quantification. As a result, 75,942 unique peptides and 8859 corresponding proteins were identified. Principal component analysis (PCA) revealed slight profile differences among these samples (Figure 4(a)). In addition, the differentially expressed proteins were identified. Compared to non‐SPA, 571 proteins in SPA samples were upregulated and 722 proteins were downregulated (Figure 4(b)). The differential proteins clearly distinguished the SPA and non‐SPA samples (Figure 4(c)). Consistent with previous results, GO analyses revealed that the differential proteins were enriched in multiple biological processes and molecular functions (Figures 4(d) and 4(e)). GSEA analyses were also implemented. Notably, the oxidative phosphorylation pathway was significantly enriched in SPA samples compared to non‐SPA (Figure 4(f)). Besides, we estimated the immune cell infiltration and correlated with oxidative phosphorylation pathway activity (estimated by ssGSEA), and found that different immune subtypes have significantly different oxidative phosphorylation pathway activities, highest in the endothelial subtype and lowest in the CD4+ T cell infiltration subtype, indicating that it also influenced or was influenced by immune infiltration (Figure S2). In addition, we also analyzed the transcriptome of GSE147786, which contains 22 non‐SPAs and 22 SPAs. Totally, 42 out of 101 oxidative phosphorylation–related genes were identified as differentially expressed (Table S2), consistent with previous results.

Figure 4Validation of oxidative phosphorylation pathway in additional pituitary adenoma samples. (a) Principal component analysis (PCA) of proteomic data from validation samples. (b) Differentially expressed proteins in the validation dataset. (c) Clustering of validation samples based on differential protein expression. (d) Enriched Gene Ontology (biological process) terms in differential proteins. (e) Enriched Gene Ontology (molecular function) terms in differential proteins. (f) GSEA showing enrichment of the oxidative phosphorylation pathway in SPA samples. (g) Hormone secretion quantification after AICAR and Rotenone treatment using ELISA in HP75 and GH3 cell lines.

**Figure:**
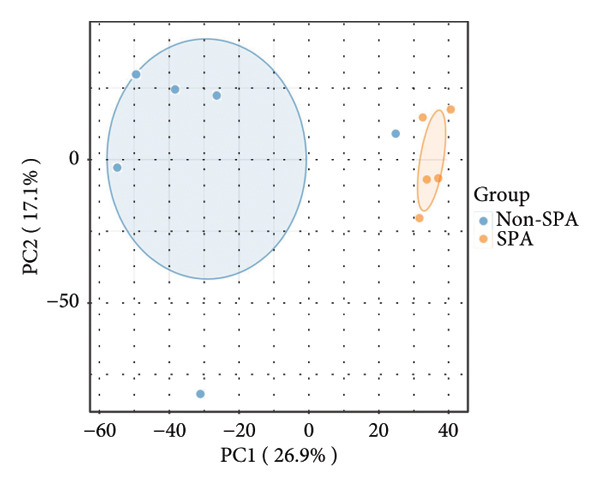
(a)

**Figure:**
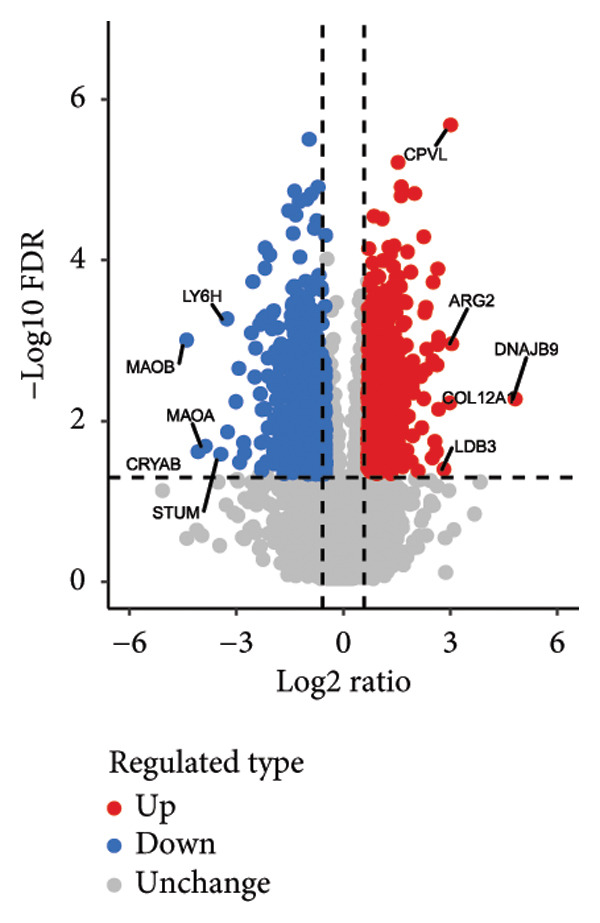
(b)

**Figure:**
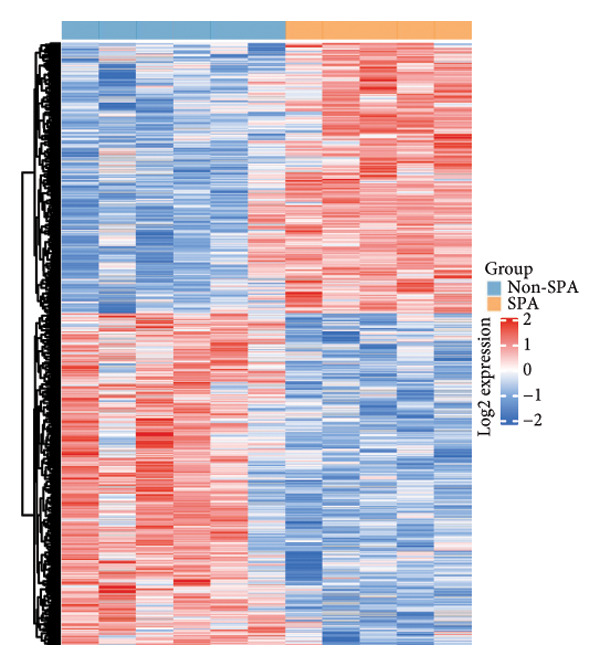
(c)

**Figure:**
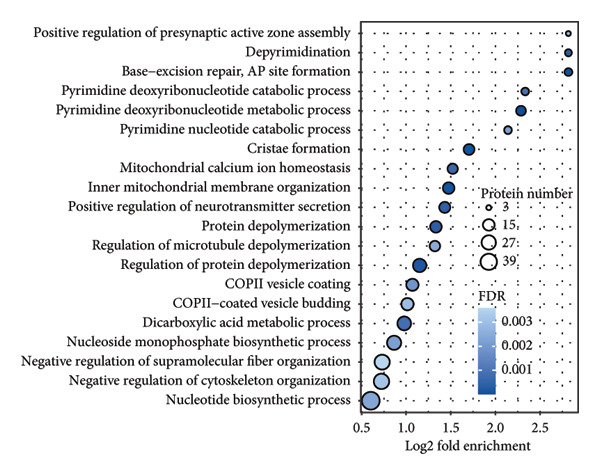
(d)

**Figure:**
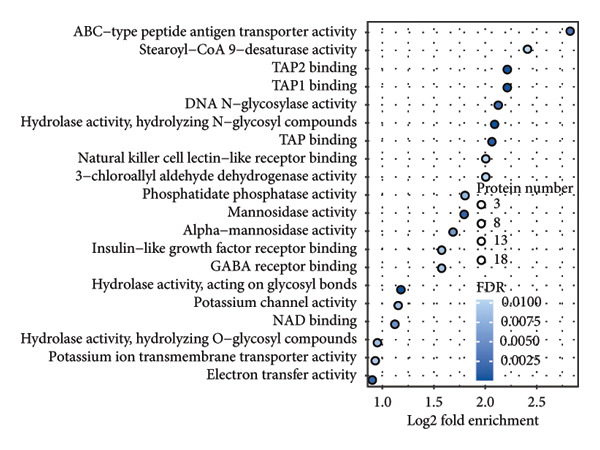
(e)

**Figure:**
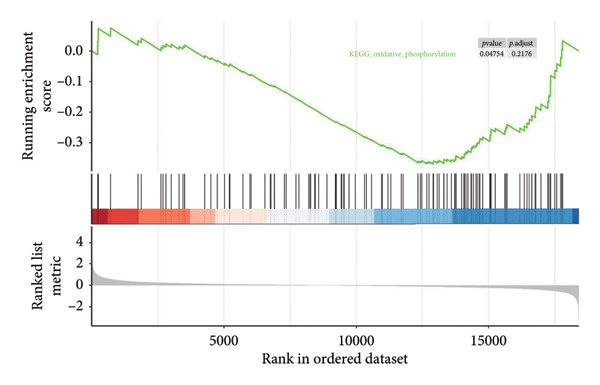
(f)

**Figure:**
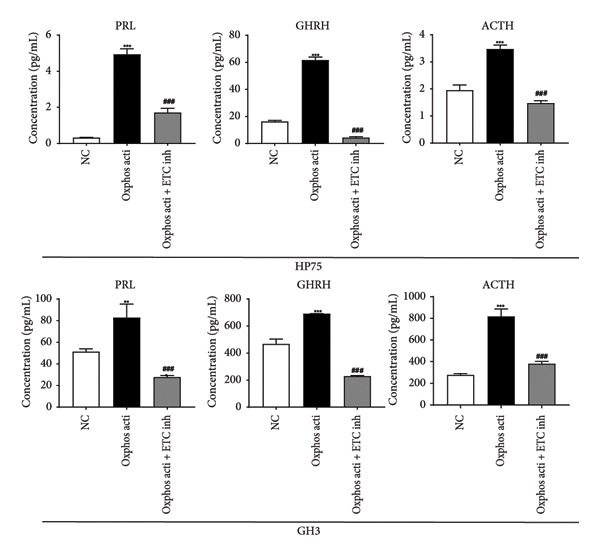
(g)

To further validate the role of oxidative phosphorylation in hormone secretion, we obtained SPA and non‐SPA cell lines from human and rat (HP75, purchased as a SPA cell line that originated from human, and GH3, a primary cell line that originated from rat). After adding oxidative phosphorylation activator AICAR, the secretion of hormones (including adrenocorticotropic hormone [ACTH], growth hormone–releasing hormone [GHRH], and PRL), determined by enzyme‐linked immunosorbent assay (ELISA), was significantly elevated (Figure 4(g)). Meanwhile, the electron transfer chain (ETC) inhibitor Rotenone restored this. Taken together, oxidative phosphorylation alteration significantly changed the hormone secretion ability of pituitary adenoma cells.

### 3.3. Protein–Protein Interaction Network Revealed SPA and Non‐SPA Regulatory Differences

To comprehensively understand the biological differences between SPA and non‐SPA samples, the identified differentially expressed genes in mRNA, protein, and our own dataset were intersected, resulting in 283 unique genes (Figure 5(a)). A protein–protein network was constructed using these genes by searching for the shortest path between any two genes (Figure 5(b)). Paths longer than three steps were excluded from the network. The genes with the highest betweenness centrality were shown, along with eigenvector, closeness, and degree centrality (Figure 5(c)). MAPK1, MAPK3, IDH1, and PKM had the highest degree and betweenness values. For further verification, we knocked down PKM and MAPK1 in both HP73 and GH3 cell lines (Figure 5(d)). As expected, the expression reduction of MAPK1 and PKM in both cell lines significantly reduced hormone secretion, including ACTH, GHRH, and PRL (Figure 5(e)). Besides, the MAPK1 and PKM downregulation also altered the apoptosis rate of both cell lines (Figures 5(f) and 5(g)). These genes are involved in central metabolism, including oxidative phosphorylation, especially MAPK and PKM.

Figure 5Protein–protein interaction network analysis of SPA and non‐SPA regulatory differences. (a) Intersected genes from mRNA, protein, and validation datasets. (b) Protein–protein interaction network constructed using differentially expressed genes. (c) Network parameters showing genes with the highest betweenness, eigenvector, closeness, and degree centrality. (d) Knockdown efficiency of MAPK and PKM3 in HP75 and GH3 cell lines. (e) Hormone secretion quantification after MAPK and PKM3 knockdown in HP75 and GH3 cell lines. (f) Apoptosis rates of HP75 and GH3 cell lines before and after MAPK1/PKM knockdown. (g) Statistics of apoptosis rates of HP75 and GH3 cell lines before and after MAPK1/PKM knockdown. ^∗^ *p* < 0.05, ^∗∗^ *p* < 0.01, and ^∗∗^ *p* < 0.001.

**Figure:**
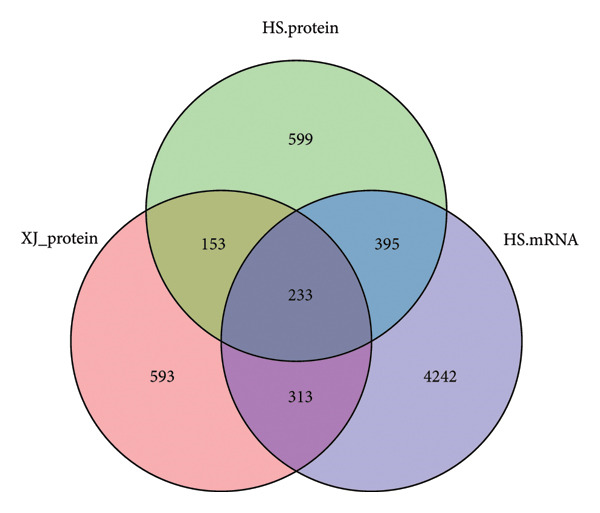
(a)

**Figure:**
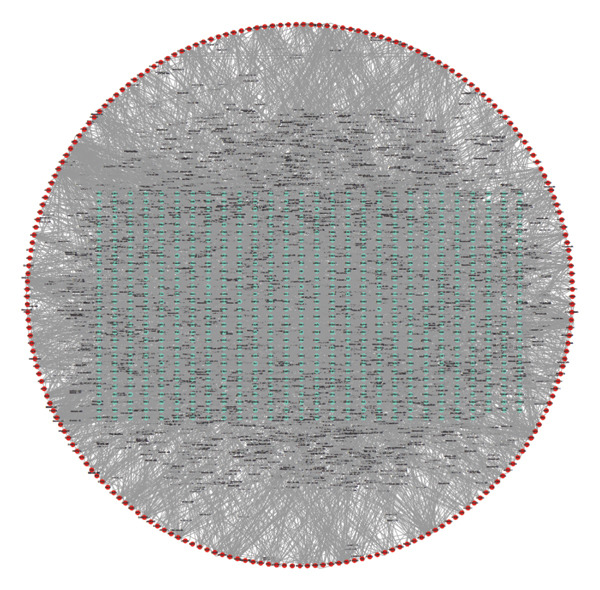
(b)

**Figure:**
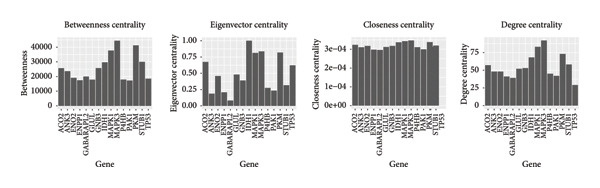
(c)

**Figure:**
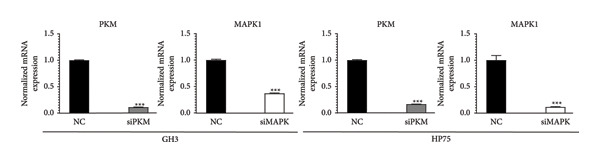
(d)

**Figure:**
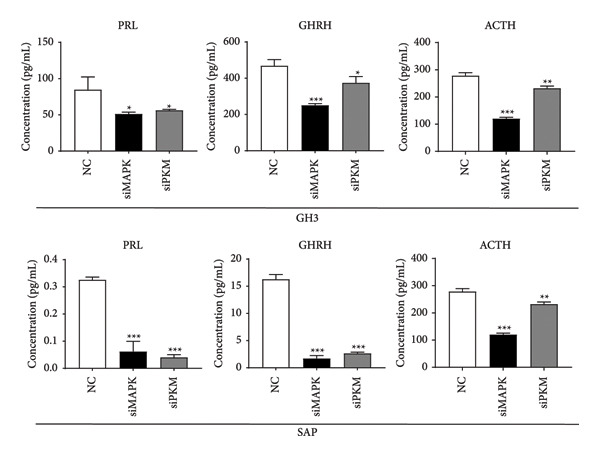
(e)

**Figure:**
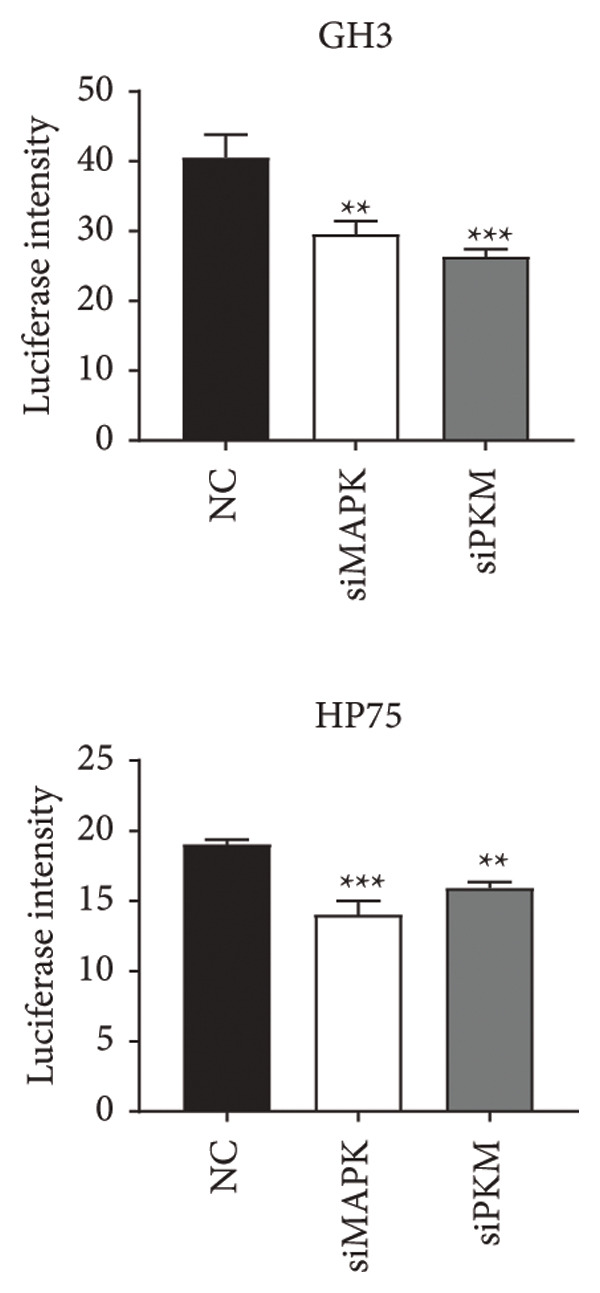
(f)

**Figure:**
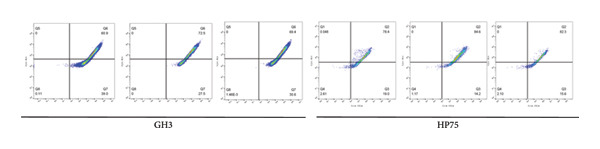
(g)

## 4. Materials and Methods

### 4.1. Sample Enrollment and Data Collection

Written consent was obtained from the patients involved, and this study was approved by the Ethics Committee of People’s Hospital of Xinjiang Uygur Autonomous Region. Patients who underwent surgery between 2018 and 2023 were enrolled in this study. Baseline information was shown in Table S1. A total of 11 patients were enrolled, seven females and four males, five SPAs and six non‐SPAs. For the HS dataset [11], mutation, gene expression, and protein abundance data were retrieved from the supporting files and processed according to standard protocols. The clinical information was also downloaded from the same website, along with the secretion information of the HS dataset.

### 4.2. Genomic Data Analyses

Mutational data in maf format were processed using the R package “maftools” [12]. Only nonsynonymous mutations, which cause alterations in the amino acid sequence, were retained for further analyses (including missense, nonsense, frameshift, splice‐site, and nonstop mutations). Copy number variation was not included in this process. The mutation landscape of SPA and non‐SPA samples was visualized with the R package “GenVisR” [13], focusing on the top 20 most frequently mutated genes. The differentially mutated genes between the SPA and non‐SPA groups were identified using Fisher’s exact test. Detailed information on amino acid mutations and the distribution of samples was visualized with “maftools” and “ggplot2,” respectively. For pathway analyses, gene sets were retrieved from the MSigDB database [14]. Samples that harbored at least one mutation in specific pathways were identified as having mutational pathways in this sample.

### 4.3. Transcriptome and Proteome Analyses

Protein abundance raw data and gene expression data of the HS dataset were downloaded, and the batch effect was removed using R packages according to the method provided. The expression differences between SPA and non‐SPA samples were identified using the R package “limma” [15]. Differentially expressed genes were identified with an adjusted *p* value of < 0.05 and a log2‐transformed fold change of > 1 or < −1. The R packages “clusterProfiler” [16] and “enrichplot” were used for Gene Ontology enrichment analyses and GSEAs, respectively, by sorting the differential gene list in order of log2‐transformed fold change. The intersected genes across different biological levels (mRNA and protein) and datasets were visualized using the R package “venn.”

### 4.4. Protein Extraction

The sample was pulverized into cell powder using liquid nitrogen and then transferred to a 5‐mL centrifuge tube. Subsequently, four volumes of lysis buffer (containing 1% Triton X‐100% and 1% protease inhibitor cocktail) were added to the cell powder. This mixture was then subjected to 3 minutes of sonication on ice using a high‐intensity ultrasonic processor (Scientz) (note: for PTM experiments, specific inhibitors such as 3 μM TSA and 50 mM NAM for acetylation, or 1% phosphatase inhibitor for phosphorylation, were also included in the lysis buffer). The remaining debris was removed by centrifugation at 12,000 g for 10 min at 4°C. The supernatant was collected, and the protein concentration was measured using a BCA kit according to the manufacturer’s instructions. The sample was then slowly mixed with TCA to a final concentration of 20% (m/v) to precipitate the protein, vortexed, and incubated for 2 h at 4°C. The precipitated protein was collected by centrifugation at 4500 × g for 5 min at 4°C, washed three times with precooled acetone, and dried for 1 min. The protein sample was redissolved in 200‐mM TEAB and ultrasonically dispersed. Trypsin was added at a 1:50 trypsin‐to‐protein mass ratio for overnight digestion. The sample was then reduced with 5 mM dithiothreitol for 30 min at 56°C and alkylated with 11 mM iodoacetamide for 15 min at room temperature in the dark. Finally, the peptides were desalted using a Strata X SPE column.

### 4.5. LC‐MS/MS

The tryptic peptides were resuspended in Solvent A and immediately introduced onto a custom‐made reversed‐phase analytical column, which measured 25 cm in length and had an inner diameter of 100 μm. The mobile phase consisted of Solvent A (0.1% formic acid and 2% acetonitrile in water) and Solvent B (0.1% formic acid in acetonitrile). The peptides were separated using the following gradient program: 0–14 min at 6%–24% Solvent B, 14–16 min at 24%–35% Solvent B, 16–18 min at 35%–80% Solvent B, and 18–20 min at 80% Solvent B. The separation was carried out with a consistent flow rate of 500 nL/min on a NanoElute UHPLC system (Bruker Daltonics). The peptides were then analyzed using a capillary source coupled with timsTOF Pro 2 mass spectrometry. An electrospray voltage of 1.75 kV was applied. Precursors and fragments were detected using the TOF detector. The timsTOF Pro operated in data‐independent acquisition parallel accumulation serial fragmentation (dia‐PASEF) mode. The full MS scan range was set from 300 to 1500, with 20 PASEF‐MS/MS scans acquired per cycle. The MS/MS scan range was from 400 to 850, with an isolation window of 7 m/z.

### 4.6. Protein–Protein Interaction Network

Protein–protein interaction data were downloaded from the STRING (16) database, with human selected as the species. To filter for high‐confidence protein–protein pairs, only interactions with a combined score above 600 were retained, while the others were discarded. A high‐confidence human protein–protein interaction network was constructed. After retrieving intersected genes from the HS‐mRNA, HS‐protein, and our own proteome data, protein–protein pairs for the gene list were generated, where each combination contained two genes, both of which were intersected genes. The path for each combination of genes was searched using Dijkstra’s algorithm to find the shortest path between the genes, employing the R package “igraph.” Paths longer than three steps were discarded. Subsequently, these interactions were used to construct the protein–protein interaction network, and “Cytoscape” [17] software was used for visualization. The visualization parameters were manually adjusted. Network parameters, including betweenness, closeness, degree, and eigenvector centrality values, were calculated using the R package “igraph” and visualized with “ggplot2.”

### 4.7. Cell Culture and siRNA

The human pituitary adenoma cell line HP75 (Cat. No. SC2274) was obtained from Yuchi Biology (Shanghai, China) and cultured in DMEM supplemented with 10% FBS and 1% penicillin‐streptomycin (P/S), while the rat pituitary tumor cell line GH3 (Cat. No. CL‐0340) was purchased from Procell (Wuhan, China) and maintained in Ham’s F‐12K medium containing 15% horse serum (HS), 2.5% FBS, and 1% P/S; both cell lines were incubated at 37°C in a humidified atmosphere with 5% CO_2_ and 95% air humidity. Cells were seeded into 6‐well plates one day prior to transfection and cultured until 70%–80% confluence was reached. Two hours before transfection, the medium was replaced with serum‐free medium. For transfection complex preparation, two solutions were prepared in EP tubes per well: solution A contained 30 pmol siRNA diluted in 150 μL serum‐free medium, gently mixed by pipetting and incubated at room temperature for 5 min under sterile conditions; solution B was prepared by diluting 5 μL lipofectamine 2000 in 150 μL serum‐free medium, followed by gentle mixing and incubation for 5 min. Solution A was then combined with solution B, mixed gently, and incubated at room temperature for 20 min. The mixture was added dropwise to the cells, which were gently swirled and incubated at 37°C. After 6 h, the medium was replaced with complete medium, and the cells were cultured for an additional 48 h. Transfection efficiency was evaluated using fluorescence microscopy 48 h post‐transfection.

## 5. ELISA

The following commercially available ELISA kits from JONLNBIO (Shanghai, China) were used in this study following the instruction provided by the manufacturer: Human ACTH ELISA Kit (Cat. No. JL12509), Rat ACTH ELISA Kit (Cat. No. JL11379), Human GHRH ELISA Kit (Cat. No. JL10808), Rat GHRH ELISA Kit (Cat. No. JL20935), Human PRL ELISA Kit (Cat. No. JL11332), and Rat PRL ELISA Kit (Cat. No. JL12505). Cell culture supernatants were analyzed using ELISA to quantify the expression levels of ACTH, GHRH, and PRL. Species‐specific commercial ELISA kits were employed according to the manufacturer’s protocols. Cell samples were centrifuged at 1000 × g for 20 min at 4 °C to obtain clear supernatants for immediate analysis. All assays were performed in precoated 96‐well plates. Standards and samples were added in duplicate (100 μL/well) and incubated at 37 °C for 1 h. After incubation, biotinylated detection antibody was added without washing, followed by another 1‐h incubation at 37 °C. Plates were washed three times with phosphate‐buffered saline, then horseradish peroxidase conjugate was added, and incubated for 30 min at 37 °C. Following five additional washes, tetramethylbenzidine substrate was added and incubated for 15 min at 37 °C, protected from light. The enzymatic reaction was terminated with the stop solution, and absorbance was immediately measured at 450 nm using a microplate reader.

### 5.1. Flow Cytometry

Cells (1–6 × 10^5^) were resuspended in 0.5 mL of culture medium, which may contain serum and phenol red, followed by the addition of 0.5‐mL JC‐1 staining working solution. After gentle inversion mixing, the cells were incubated at 37°C for 20 min. During incubation, an appropriate amount of JC‐1 staining buffer (1X) was prepared by diluting JC‐1 staining buffer (5X) with distilled water at a 1:4 ratio and kept on ice. After incubation, the cells were centrifuged at 600 × g at 4°C for 3–4 min to form a pellet, and the supernatant was carefully removed without disturbing the pellet. The cells were then washed twice with ice‐cold JC‐1 staining buffer (1X): each wash involved resuspension in 1‐mL buffer, centrifugation under the same conditions, and careful aspiration of the supernatant. Finally, the cells were resuspended in an appropriate volume of JC‐1 staining buffer (1X) and analyzed using flow cytometry.

## 6. Discussion

This study analyzed the clinical, genomic, transcriptomic, and proteomic differences between SPA and non‐SPA samples, comprehensively identifying the signatures of SPA and non‐SPA and emphasizing the role of the oxidative phosphorylation pathway. Among these signatures, we noticed that the TCHH mutation rate was significantly higher in non‐SPA samples compared to SPA samples. TCHH is known for its role in providing hair strength and structure [18]. However, recent reports have also highlighted its role in cancers. For example, Jingfang et al. reported that microsatellite instability–high colorectal cancer harbors a significantly higher TCHH mutation rate compared to microsatellite instability–low samples, and it may serve as a prognostic biomarker for colorectal cancer [19]. Consistently, DNA methylation of TCHH, estimated by cell‐free DNA, has shown its potential in predicting metastasis of colorectal cancer [20]. Despite these reports, the functions of TCHH in pituitary adenomas remain to be thoroughly investigated compared to other frequently mutated genes. Since the role of TCHH is associated with matrix formation, we suspect that the mutation of TCHH may alter the microenvironment of pituitary adenomas. Consistent with this hypothesis, we found that the GAP junction signaling pathway was also significantly differentially mutated: the mutation rate of GAP junction signaling was 24% in non‐SPA samples, while only 12% of SPA samples exhibited mutations. The impact of the microenvironment on pituitary adenomas has long been discussed [21, 22]. Nonetheless, this hypothesis requires further investigation.

The Warburg effect has been known for nearly a hundred years and refers to the higher glucose consumption by converting it to lactate instead of utilizing oxidative phosphorylation [23]. Despite most cancer cells retaining mitochondrial respiration, glucose is still diverted away from the high‐energy–yielding pathway (oxidative phosphorylation) and switched to an energy‐waste pathway. In this study, we detected that oxidative phosphorylation pathways were enriched in non‐SPA samples compared to SPA samples. As we know, cancer originates from different tissues or cell types [24], and these cancers retain the phenotypes of their origin. Thus, we suspect that the retained oxidative phosphorylation is a marker for cancer differentiation [25], which results in the loss of the original tissue phenotypes′ functions.

There are several limitations to this study. First of all, this is a retrospective study. Although we collected samples from our affiliation, most of the samples were previously released data. Detailed clinical information is missing, making it difficult to estimate the impact of these samples accurately. Second, the results and conclusions need further validation. For example, the roles of TCHH mutation and oxidative phosphorylation loss in pituitary adenoma secretion have yet to be validated. Lastly, the number of validation samples is limited, which may introduce bias in estimating the role of oxidative phosphorylation.

## 7. Conclusion

This study offers new insights into the biological mechanisms underlying pituitary adenomas and provides valuable directions for future research, emphasizing the importance of oxidative phosphorylation in tumor behavior and potential therapeutic targets.

## Conflicts of Interest

The authors declare no conflicts of interest.

## Author Contributions

Yuan Chen and Qiang Zhao contributed equally to this work.

## Funding

This study was funded by the Fund Project of the Nature Fund of Xinjiang Uygur Autonomous region (2022D01C619) and the Fund Project of the People’s Hospital of Xinjiang Uygur Autonomous Region (20230307).

## Supporting Information

Additional supporting information can be found online in the Supporting Information section.

## Supporting information


**Supporting Information 1** Table S1: Baseline information of enrolled samples.

**Supporting Information 2** Table S2: Differentially expressed genes in GSE147786.

**Supporting Information 3** Figure S1: Infiltration of cells correlated with the related TCHH mutation.

**Supporting Information 4** Figure S2: Immune subtypes correlated with the related TCHH mutation.

## Data Availability

The data that support the findings of this study are available on request from the corresponding author. The data are not publicly available due to privacy or ethical restrictions.
