# Supplementary material for: Oxidative Phosphorylation in Silent Pituitary Adenomas: A Multiomics Perspective
Source: Int J Endocrinol. 2026 Jan 28;2026:8488950. doi: 10.1155/ije/8488950 (PMC12849212; doi:10.1155/ije/8488950)
Supplement: Supplementary file 3 — Supporting Information 3 Figure S1: Infiltration of cells correlated with the related TCHH mutation. [file IJE-2026-8488950-s003.pdf]

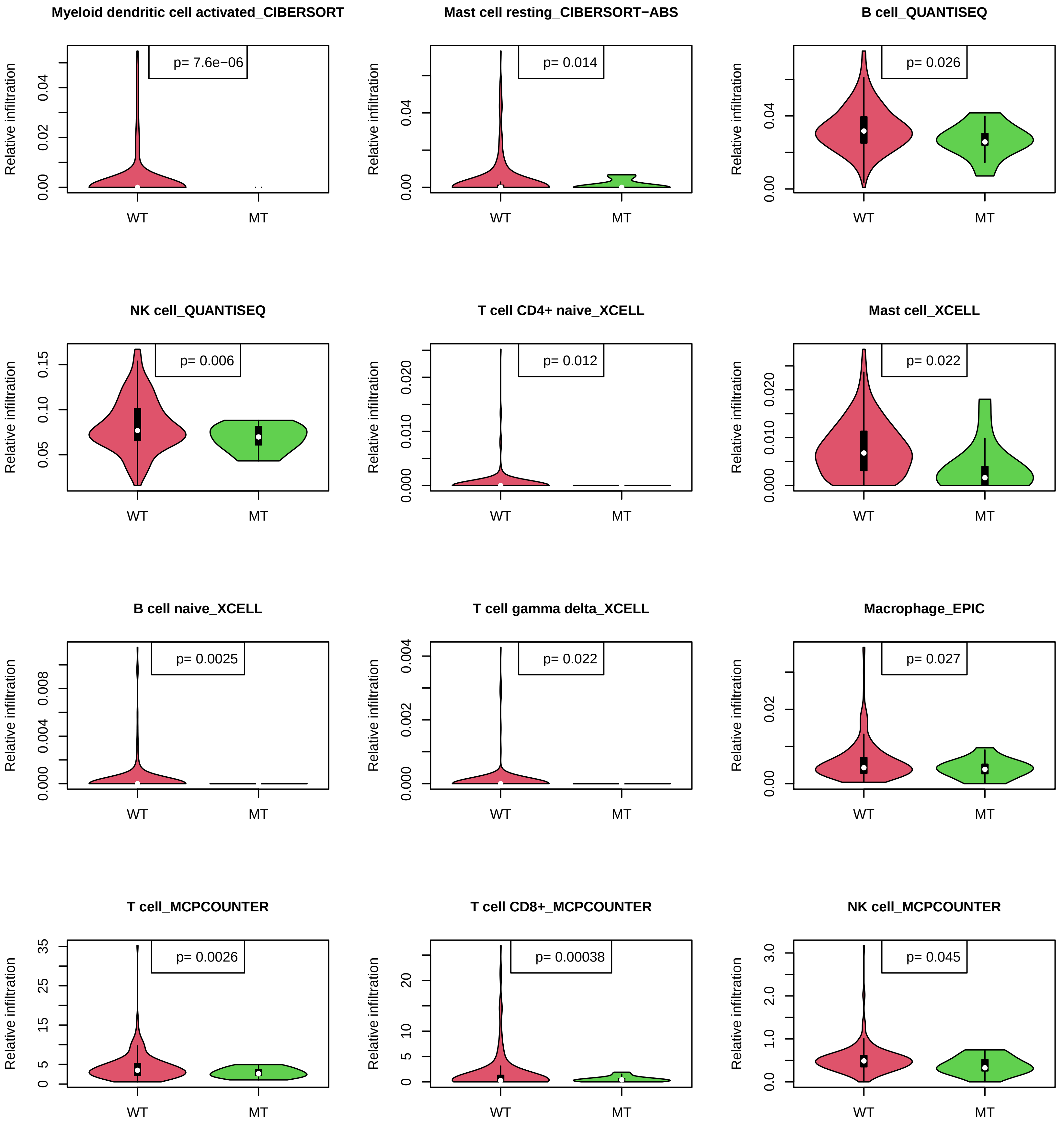

Myeloid dendritic cell activated_CIBERSORT
p= 7.6e−06
Mast cell resting_CIBERSORT−ABS
p= 0.014
B cell_QUANTISEQ
p= 0.026
NK cell_QUANTISEQ
p= 0.006
T cell CD4+ naive_XCELL
p= 0.012
Mast cell_XCELL
p= 0.022
B cell naive_XCELL
p= 0.0025
T cell gamma delta_XCELL
p= 0.022
Macrophage_EPIC
p= 0.027
T cell_MCPCOUNTER
p= 0.0026
T cell CD8+_MCPCOUNTER
p= 0.00038
NK cell_MCPCOUNTER
p= 0.045
Relative infiltration
WT
MT
