# Supplementary figures and images for: Oxidative Phosphorylation in Silent Pituitary Adenomas: A Multiomics Perspective
Source: Int J Endocrinol. 2026 Jan 28;2026:8488950. doi: 10.1155/ije/8488950 (PMC12849212; doi:10.1155/ije/8488950)

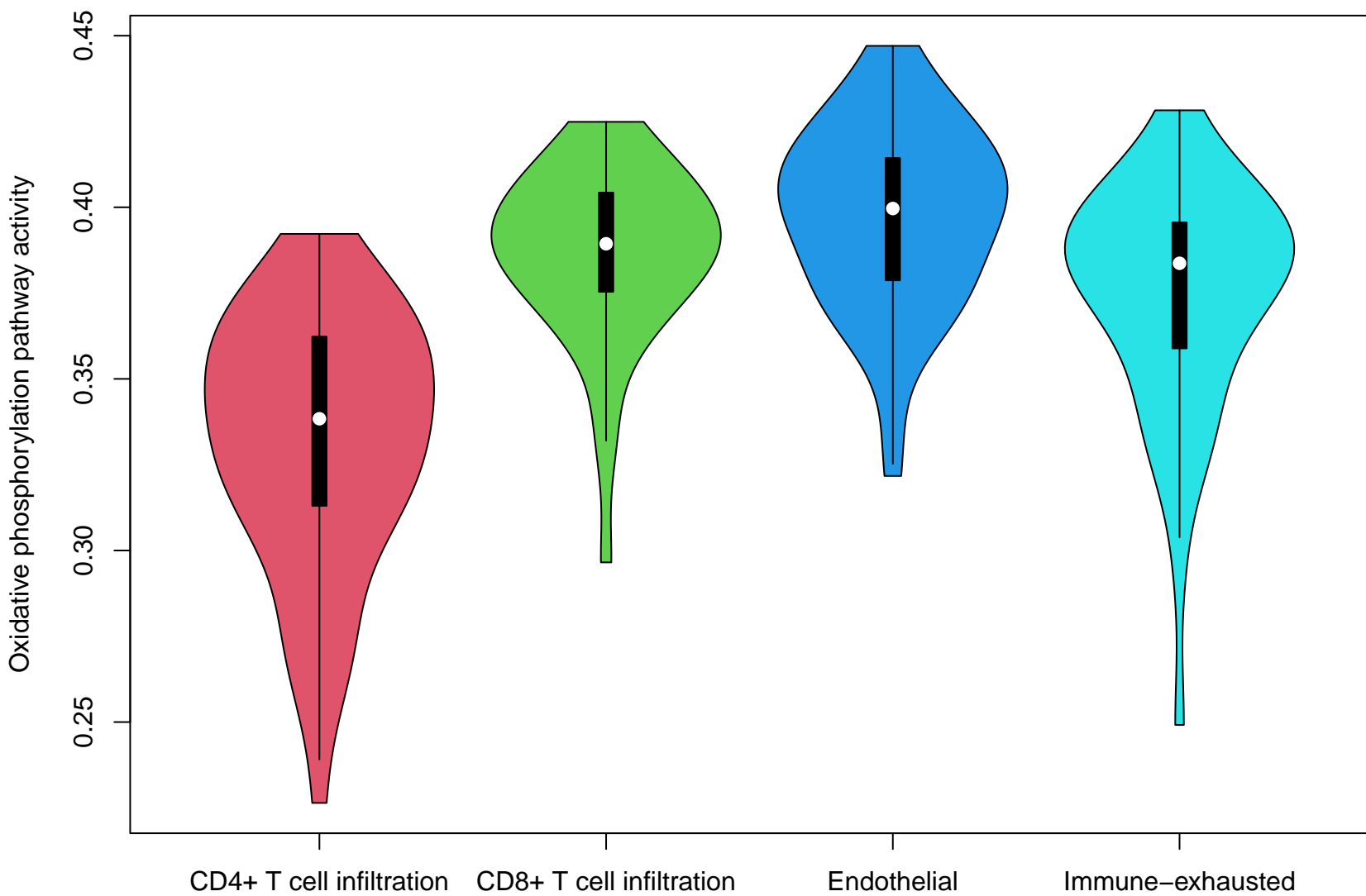

Supplement: Supplementary file 4 — Supporting Information 4 Figure S2: Immune subtypes correlated with the related TCHH mutation. [file IJE-2026-8488950-s002.pdf]
